# An Enhanced Transfer Learning Based Classification for Diagnosis of Skin Cancer

**DOI:** 10.3390/diagnostics12071628

**Published:** 2022-07-05

**Authors:** Vatsala Anand, Sheifali Gupta, Ayman Altameem, Soumya Ranjan Nayak, Ramesh Chandra Poonia, Abdul Khader Jilani Saudagar

**Affiliations:** 1 Institute of Engineering and Technology, Chitkara University, Rajpura 140401, Punjab, India; vatsala.anand@chitkara.edu.in (V.A.); sheifali.gupta@chitkara.edu.in (S.G.); 2 Department of Computer Science and Engineering, College of Applied Studies and Community Services, King Saud University, Riyadh 11533, Saudi Arabia; aaltameem@ksu.edu.sa; 3 Amity School of Engineering and Technology, Amity University Uttar Pradesh, Noida 201301, Uttar Pradesh, India; nayak.soumya17@gmail.com; 4 Department of Computer Science, CHRIST (Deemed to be University), Bangalore 560029, Karnataka, India; rameshcpoonia@gmail.com; 5 Information Systems Department, Imam Mohammad Ibn Saud Islamic University (IMSIU), Riyadh 11432, Saudi Arabia

**Keywords:** skin cancer, Kaggle, convolutional neural network, benign, malignant, data augmentation, VGG16, classification

## Abstract

Skin cancer is the most commonly diagnosed and reported malignancy worldwide. To reduce the death rate from cancer, it is essential to diagnose skin cancer at a benign stage as soon as possible. To save lives, an automated system that can detect skin cancer in its earliest stages is necessary. For the diagnosis of skin cancer, various researchers have performed tasks using deep learning and transfer learning models. However, the existing literature is limited in terms of its accuracy and its troublesome and time-consuming process. As a result, it is critical to design an automatic system that can deliver a fast judgment and considerably reduce mistakes in diagnosis. In this work, a deep learning-based model has been designed for the identification of skin cancer at benign and malignant stages using the concept of transfer learning approach. For this, a pre-trained VGG16 model is improved by adding one flatten layer, two dense layers with activation function (LeakyReLU) and another dense layer with activation function (sigmoid) to enhance the accuracy of this model. This proposed model is evaluated on a dataset obtained from Kaggle. The techniques of data augmentation are applied in order to enhance the random-ness among the input dataset for model stability. The proposed model has been validated by considering several useful hyper parameters such as different batch sizes of 8, 16, 32, 64, and 128; different epochs and optimizers. The proposed model is working best with an overall accuracy of 89.09% on 128 batch size with the Adam optimizer and 10 epochs and outperforms state-of-the-art techniques. This model will help dermatologists in the early diagnosis of skin cancers.

## 1. Introduction

Cells are the basic building blocks in the human body and are used for building tissues. The skin acts as the outer layer of human beings and shields the human body against infections and harmful radiation. The three different layers of skin are the inner-most dermis, middle hypodermis, and outer epidermis. Skin cancer is the most frequently diagnosed cancer, and the occurrence of skin cancer is growing. Skin cancer is caused by the unusual development of cells. People having fair skin are prone to skin cancer. The two types of skin cancer are malignant and benign [1]. Cancerous cells that grow without any control leads to malignant tumors. Metastasis is a process in which the cancer cells travel through the lymph nodes or circulation and extend to further parts of the human body. According to the report of the American Cancer Society (ACS), in 2018, 13,460 skin cancer death cases were noted [2,3]. Throughout the years, detecting melanomas via image analysis has shown progression. Most studies of melanomas were based on machine learning algorithms. However, deep learning algorithms have helped in skin lesion classification studies [4].

Haenssle et al. [5] utilized a CNN for classification of dermatoscopy melanocytic images and obtained a value of sensitivity and a specificity as 86.6%. Dorj et al. [6] used an approach of ECOC SVM with an AlexNet pre-trained model for multi-class skin disease classification. They obtained an accuracy of 95.1%. Han et al. [7] used a deep convolutional neural network on 12 different skin diseases. They obtained an accuracy of 96.0%. Khan et al. [8] used various pre-trained architectures like VGG16, DenseNet169, DenseNet161, and ResNet50. In this, they pushed the boundary of neural networks by using low resolution pixels such as 80 × 80, 64 × 64, and 32 × 32. They achieved the highest performance values of 80.46%, 78.56%, and 74.15% for 80 × 80, 64 × 64, and 32 × 32 pixels, respectively. Mohakud et al. [9] has proposed an encoder decoder network for segmentation of image. The authors obtained the value of the Jaccard coefficient as 96.41% and 86.85% respectively, and the Dice coefficient as 98.48% and 87.23%, accuracy as 98.32% and 95.25% respectively for ISIC 2016 and ISIC 2017 dataset.

Agrahari et al. [10] used a pre-trained MobileNet model for building the model and worked using HAM10000 dataset. They obtained categorical accuracy as high as 80.81%. Although Chaturvedi et al. [11] had achieved an overall accuracy of 83.1% using MobileNet architecture with HAM10000 dataset. Hosny et al. [12] used AlexNet model by replacing the last layer by softmax for classification of three skin diseases. They worked on Ph2 dataset and obtained values of accuracy, sensitivity, specificity, and precision as 98.61%, 98.33%, 98.93%, and 97.73%, respectively. Abdar et al. [13] used three uncertainty quantification methods. The accuracy obtained by the model was 88.95%. Fujisawa et al. [14] used 4867 clinical images including benign and malignant conditions. The overall accuracy of the model was 76.5% with sensitivity of 96.3% and specificity of 89.5%. Garcia Arroyo et al. [15] presented an algorithm based on machine learning and used 875 dermoscopic images. The images were collected from the Interactive Atlas of Dermoscopy dataset. Total achieved accuracy was 88.00%, sensitivity was 83.44% and specificity was 90.71%. Iyatomi et al. [16] took 213 dermoscopic images and used a linear classifier. They achieved specificity of 95.90%, and the value of the area under the curve was 0.993. In 2018, Chatterjee et al. [17] used classifiers and took 4094 skin cancer images and accomplished an accuracy of 98.28% and a sensitivity of 97.63%. In 2019, these authors had taken dermoscopic images from the internet and performed GLCM and FRTA feature extraction. They attained 97.54% accuracy [18]. Gonzalez et al. [19] applied the DermaKNet technique on a total of 2750 skin cancer images and achieved an area under the curve value of 91.7%. In 2018, Ka-wahara et al. [20] applied Multi-task multi-modal neural nets on 1011 dermoscopic images. The architecture was able to localize discriminate information and also produce feature vectors.

Koohbanani et al. [21] in 2018 used transfer learning based model and used a total of 2594 dermoscopic images from the internet. This framework incorporates a variant of UNet architecture. Filali et al. [22] presented a network based on CNN by using 1000 images from the internet and realized 93.50% accuracy. Kadampur et al. [23] applied a technique based on machine learning with 104 images of skin from the internet. They realized 86.00% value of sensitivity and 73.00% specificity. For increasing deep learning performance for melanoma screening, Menegola et al. [24] presented a transfer method. A pre-trained model for detecting Diabetic Retinopathy was proposed in this study which was based on Kaggle Challenge [25].

The deep learning revolution has played a great role, with the suggestion of better architectures of the convolutional neural network [26,27,28,29,30,31]. In the following paper, a model is presented to classify skin cancer with the help of dermoscopy images. The presented model is evaluated on ten and twenty epochs using the Adam optimizer and batch size values of 8, 16, 32, 64, and 128. The proposed model has presented favorable results that will work as another estimation tool for dermatologists. The major contributions of this study include:

1. A paradigm based on transfer learning has been presented using VGG16 architecture for classification of skin cancer into benign and malignant [32];
2. The VGG16 model has been improved by the addition of one flatten layer, two dense layers with activation function (LeakyReLU) and another dense layer with activation function (sigmoid) to improve the accuracy of the model;
3. The data augmentation techniques have been performed in the pre-processing stage for increasing randomness and dataset count in order to provide stability to the proposed model;
4. The efficacy of the proposed model is achieved by analyzing various hyper parameters such as batch size, epochs, and optimizer.

The rest of the paper is arranged as follows: Section 2 shows the proposed framework model followed by results and discussions in Section 3 and conclusion in Section 4.

## 2. Proposed Framework Model

**Figure 1:**
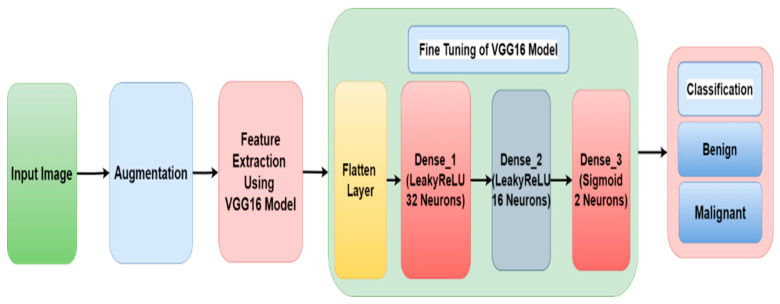
Proposed Framework Model.

A paradigm based on transfer learning has been improved and changed for classification of skin cancer into benign and malignant class. The training and testing of the model is performed on the Kaggle dataset [25] that consists of 3297 skin cancer images. The block diagram of the proposed framework model is shown in Figure 1.

### 2.1. Input Dataset

**Figure 2:**
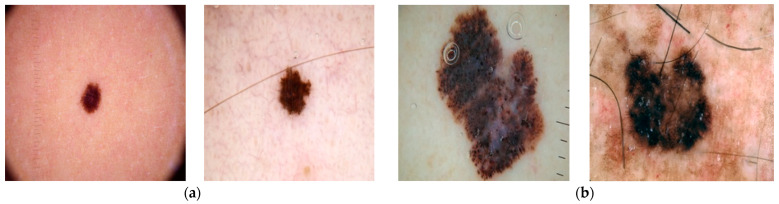
(**a**) Benign, (**b**) Malignant.

The database which is used in the study consists of 3297 skin cancer images that are collected from the Kaggle database [25]. It is comprised of RGB images of 1800 benign and 1497 malignant images of dimensions (224 × 224 × 3) pixels. Figure 2 shows a sample of benign and malignant skin cancer images from the database.

Table 1 shows the dataset description in which the number of training images, testing images and validation images are shown for both skin cancer classes. Total images in the dataset are 3297 out of which 1800 are benign and 1497 are malignant. The dataset is split into testing, training and validation. For the testing purpose, almost 10% of benign and malignant images are used. From the remaining images, 5% of the images are used for validation purposes. The remaining dataset is used for training the model.

### 2.2. Data Augmentation

**Figure 3:**
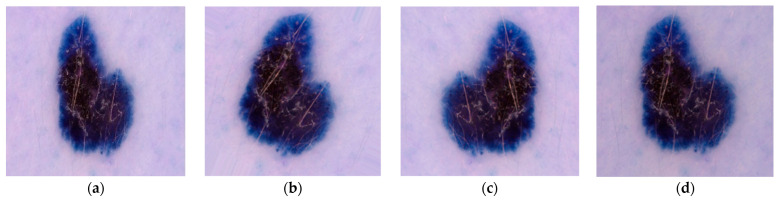
Data Augmentation Techniques in Sequence (**a**) Input Image, (**b**) Rotated Image, (**c**) Flipped Image, (**d**) Brightened Image.

A huge quantity of dataset is essential to attain the best accuracy in the DL. The augmentation of data is done with various transformation techniques [33,34,35] like rotation, flipping, and brightening in sequence as shown in Figure 3. For this, the input image is rotated 90 degrees in a clockwise direction. After that, the rotated image is flipped horizontally as well as vertically. At the end, the brightness level of the flipped image is changed by 0.8. The augmentation process is applied only on training images to train the model more precisely. In this way, the number of training images is doubled from 2818 to 5636.

Table 2 shows the total images of training, testing and validation data after augmentation. The augmentation is applied only on the training images. Previously, the training images of benign and malignant are 1534 and 1284, respectively. After the augmentation, there was a total of 5636 training images.

### 2.3. Feature Extraction Using VGG16

**Figure 4:**
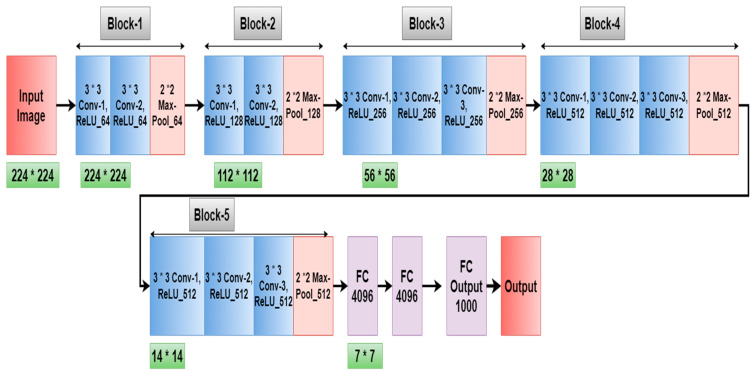
VGG16 Architecture.

We found that the majority of biomedical imaging transfer learning methods used VGG approaches to achieve the highest levels of prediction accuracy after thoroughly analyzing the methodologies. The authors of this work are inspired to implement VGG16 [32] by hyper-tuning the parameters in order to achieve the maximum possible accuracy. This is a deep Convolutional Neural Network (CNN) architecture with several16 layers, known as VGG16. In ImageNet, the VGG16 model performs about 92.7 percent of the top-five tests correctly. There are more than 14 million photos in ImageNet [36], which can be divided into more than 1000 categories. It was also one of the most popular models submitted to the 2014 International Laser Sintering and Research Conference. In the VGG16 architecture, an input image with 224 * 224 size is applied as shown in Figure 4. The VGG16 architecture consists of five blocks. In the first and second block, two convolution layers (3 * 3), and one max pooling layer (2 * 2) are applied with 64 and 128 filters, respectively. In the third, fourth, and fifth blocks, three convolution layers with 256, 512, and 512 filters are used, respectively, followed by a max pool layer (2 * 2). Therefore, in the proposed work, the VGG16 model is further modified by adding one flatten layer, two dense layers with LeakyReLU activation function, and another dense layer with activation function (sigmoid) to enhance the accuracy of this model.

Table 3 shows the images after filtration with each block after every conv layer and max-pool layer. Block 1 and block 2 consist of two conv layers and one max-pool layer. Hence the images of the 3rd convolution layer are not shown in block 1 and block 2. For each layer only a single filtered image is shown in the table. For example, in convolution layer 1 of block 1, 64 filters are used, so 64 filtered images will be received after that convolution layer.

### 2.4. Fine Tuning of VGG16 Model

**Figure 5:**
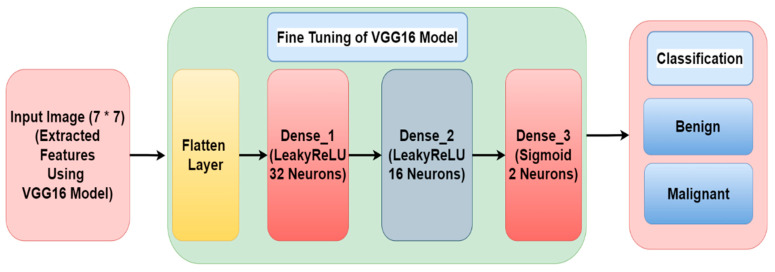
Fine Tuning of VGG16 Model.

Figure 5 displays the fine tuning of the VGG16 model. Extracted features from the VGG16 model are provided as input to the flatten layer. After that, it is transferred to the two dense layers having 32 and 16 neurons, respectively, with LeakyReLU as the activation function. The third dense layer consists of two neurons and a sigmoid activation function. After that, the image is classified into one of two different classes of cancer (i.e., benign and malignant).

Table 4 shows the parameters of the proposed model. The output image size after the VGG16 model is 7 * 7 and the number of parameters is 1,471,468. After VGG16, the flatten layer is added whose output shape is 25,088 * 1. After that, different layers like Dense_1, Dense_2 and Dense_3 are added having 802, 848, 528, and 17 layers, respectively. LeakyReLU and Sigmoid activation functions are used for these dense layers.

## 3. Results and Discussion

This contains all of the outcomes obtained using the proposed model. On the Kaggle dataset, the model is tested. Various performance criteria such as precision, sensitivity, F1 Score, and accuracy are taken into account when analyzing the suggested model. An exploratory investigation is carried out using various hyper parameters, which are described in detail below.

### 3.1. Hyper Parameters Tuning

Different parameters such as optimizer [37], batch size and epochs are used for hyper parameters tuning on dermoscopy images. The Adam optimizer is a frequently used optimizer that has replaced the Stochastic Gradient Descent optimizer in terms of training the deep learning algorithms. Adam combines the various characteristics of RMSProp and AdaGrad optimizers. The expression of Adam optimizer is given in Equations (1) and (2):

(1) $$p_{t}=\alpha_{1}p_{t-1}+\left(1-\alpha_{1}\right)\left[\frac{\delta L}{\delta \;w_{t}}\right]\;$$

(2) $$q_{t}=\alpha_{2}q_{t-1}+\left(1-\alpha_{2}\right){\left[\frac{\delta L}{\delta \;w_{t}}\right]}^{2}$$

*α*_1_ and *α*_2_ are the decay rates, *δL* is loss function derivative, *δw_t_* is weights derivative at *t*, *wt* signifies the weights, *p_t_* is gradients collection, and *q_t_* is past gradients sum of squares. Batch size is the most significant hyper parameter that is used for tuning any deep learning system. A large batch size causes the computational speedups during training of a deep learning model because of parallelism of GPUs but it may cause poor generalization. A small batch size causes faster convergence to good solutions. Hence there is always a competition between large and small batch size. In this paper, the proposed model is simulated with different batch sizes like 8, 16, 32, 64, and 128 to analyze which batch size will be suitable for better accuracy.

Epoch is the overall amount of times the whole dataset is received by neural network. When a model is trained for one epoch, it means that training dataset had one chance to update the internal parameters of the model. Therefore, the number of epochs should be more so that error can be minimized during learning of the model. But more epochs increase the computational time period. Hence, there should be a trade-off between a high and a small number of epochs. In this paper, the presented model is simulated using 10 and 20 epochs. Table 5 shows the name of hyper tuning parameters and their values.

### 3.2. Model Accuracy and Model Loss Analysis

**Figure 6:**
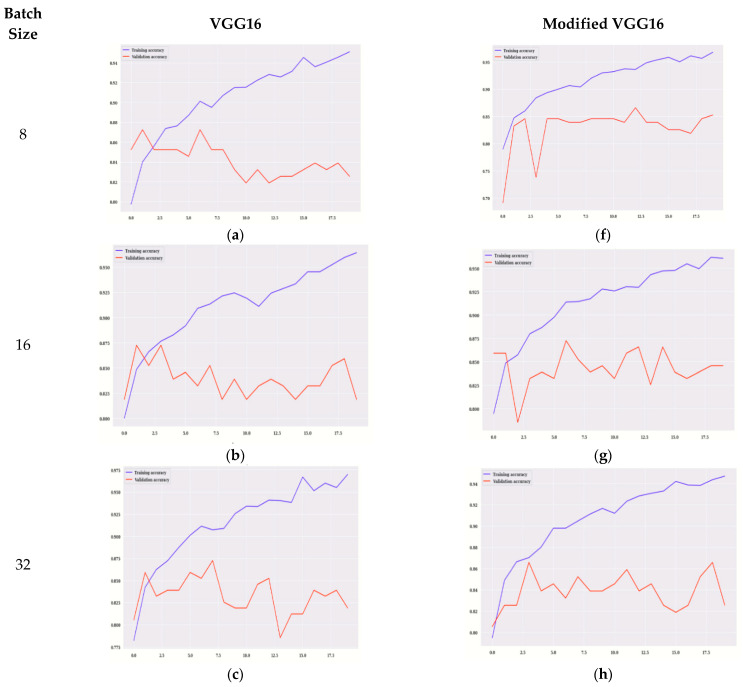

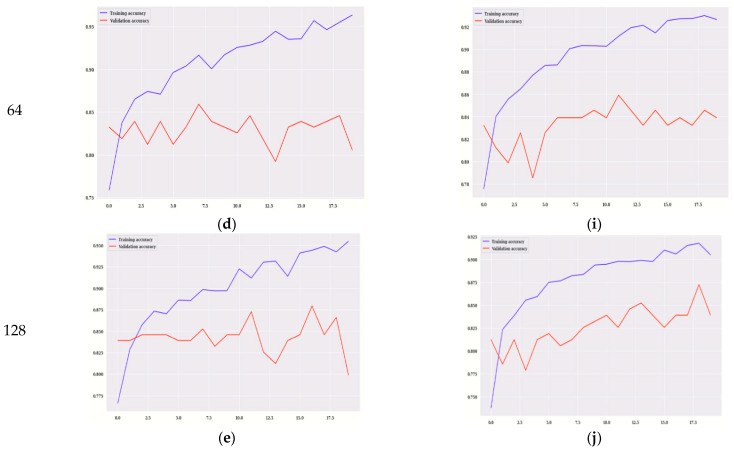
Training Accuracy for VGG16 on Batch size (**a**) 8 (**b**) 16 (**c**) 32 (**d**) 64 (**e**) 128, Training Accuracy for Modified VGG16 on Batch size (**f**) 8 (**g**) 16 (**h**) 32 (**i**) 64 (**j**) 128.

Training accuracy, validation accuracy for VGG16 and modified VGG16 is performed on the basis of model accuracy and model loss Figure 6 displays the graphs of training accuracy, validation accuracy for VGG16, and modified VGG16. 

Figure 6a–e displays the accuracy for VGG16 and Figure 6f–j displays the training, validation accuracy of modified VGG16. The model is evaluated on 20 epochs. Figure 6a,f shows the graph of training and validation accuracy on 8 batch size for VGG16 and the modified VGG16 model, respectively.

It is observed that, for modified VGG16, the values of validation accuracy are more in comparison to the VGG16 model. The highest value is on the 11th epoch that is approximately 87% for the modified VGG16 model.

Figure 6b,g shows the graph of validation and training accuracy on 16 batch size for VGG16 and the modified VGG16 model, respectively. It is observed that, for modified VGG16, the values of validation accuracy are more in comparison to VGG16 model. The highest value is on the 6th epoch that is approximately 87% for the modified VGG16 model.

Figure 6c,h shows the graph of validation and training accuracy on 32 batch size for VGG16 and the modified VGG16 model, respectively. It is observed that, for modified VGG16, the values of validation accuracy are more in comparison to the VGG16 model. The highest value is on the 3rd and 18th epoch that is approximately 86% for the modified VGG16 model.

Figure 6d,i shows the graph of validation and training accuracy on 64 batch size for VGG16 and the modified VGG16 model, respectively. It is observed that, for modified VGG16, the values of validation accuracy are more in comparison to the VGG16 model. The highest value is on the 11th epoch that is 86% for the modified VGG16 model.

Figure 6e,j shows the graph of validation and training accuracy on 128 batch size for VGG16 and the modified VGG16 model, respectively. It is observed that, for modified VGG16, the values of validation accuracy are more in comparison to the VGG16 model. The highest value is on the 18th epoch that is approximately 87.5% for the modified VGG16 model. It can be analyzed from Figure 6 that, for all the batch sizes as well as each epoch, validation accuracy is better for modified VGG16 as compared to VGG16. In any deep learning model, training and validation loss reduces as the number of epochs increases. Starting from 0 to 20 epoch values, peak points are shown in all the figures.

**Figure 7:**
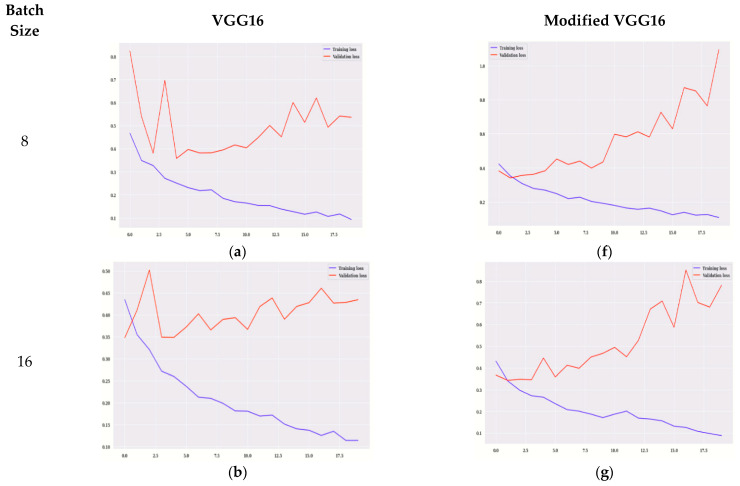

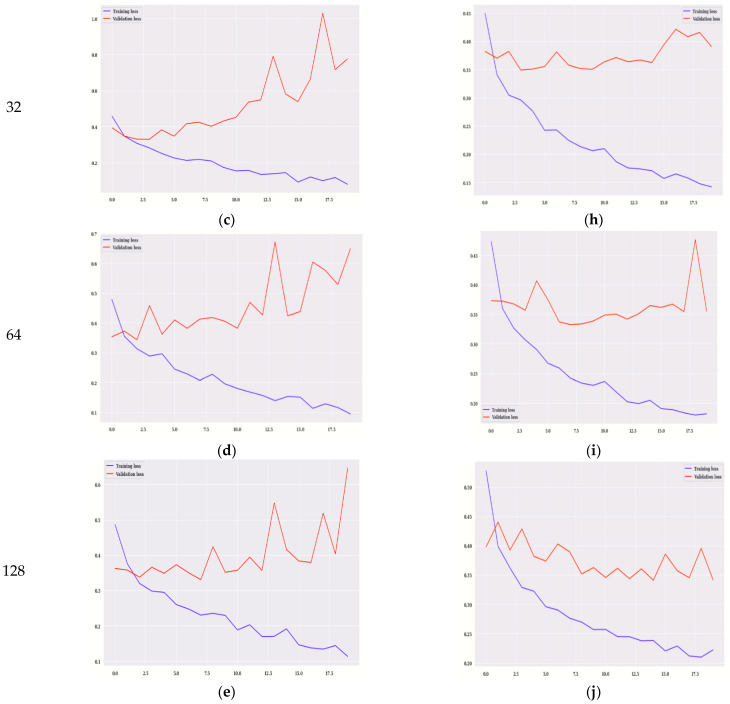
Training Loss for VGG16 on Batch Size (**a**) 8 (**b**) 16 (**c**) 32 (**d**) 64 (**e**) 128, Training Loss for Modified VGG16 (**f**) 8 (**g**) 16 (**h**) 32 (**i**) 64 (**j**) 128.

Figure 7 shows the graphs of loss for VGG16 and modified VGG16. Figure 7a–e shows the training loss for VGG16, and Figure 7f–j shows the training loss, validation loss for modified VGG16. The values of training loss are compared to validation loss. The model is evaluated on 20 epochs. Figure 7a,f shows the graph of training and validation loss on 8 batch size for VGG16 and the modified VGG16 model, respectively. It is observed that, for modified VGG16, the values of validation loss are less in comparison to the VGG16 model. Similarly, for all the batch sizes modified VGG16 is showing better results as compared to VGG16 in terms of validation loss. Starting from 0 to 20 epoch values, peak points are shown in all the figures.

Table 6 shows the loss and accuracy for training and validation of the modified VGG16 model for two different epoch values, 10 and 20. The model is simulated with the Adam optimizer at five different batch sizes (i.e., 8, 16, 32, 64, and 128). It can be seen that, on the 10th epoch, the training accuracy is maximum at batch size 32, which is 0.9255 whereas training loss is minimum (i.e., 0.1736). Whereas, on the same epoch value, validation accuracy is maximum (i.e., 84.56%), and the validation loss is less (i.e., 0.3514) for batch size 32. From Table 6, it is also analyzed that, on 20th epoch, the value of training accuracy is maximum at batch size 32, which is 0.9698, whereas training loss is minimum (i.e., 0.0801). Whereas, on the same epoch, value validation accuracy is maximum (i.e., 82.55%) at batch size 8, and the validation loss is minimum (i.e., 0.6468) at batch size 128.

Overall, it can be concluded from this table that, on the 10th and 20th epoch, the training results are best at batch size 32, whereas, validation results are best on the 10th epoch at batch size 128.

### 3.3. Confusion Matrix

**Figure 8:**
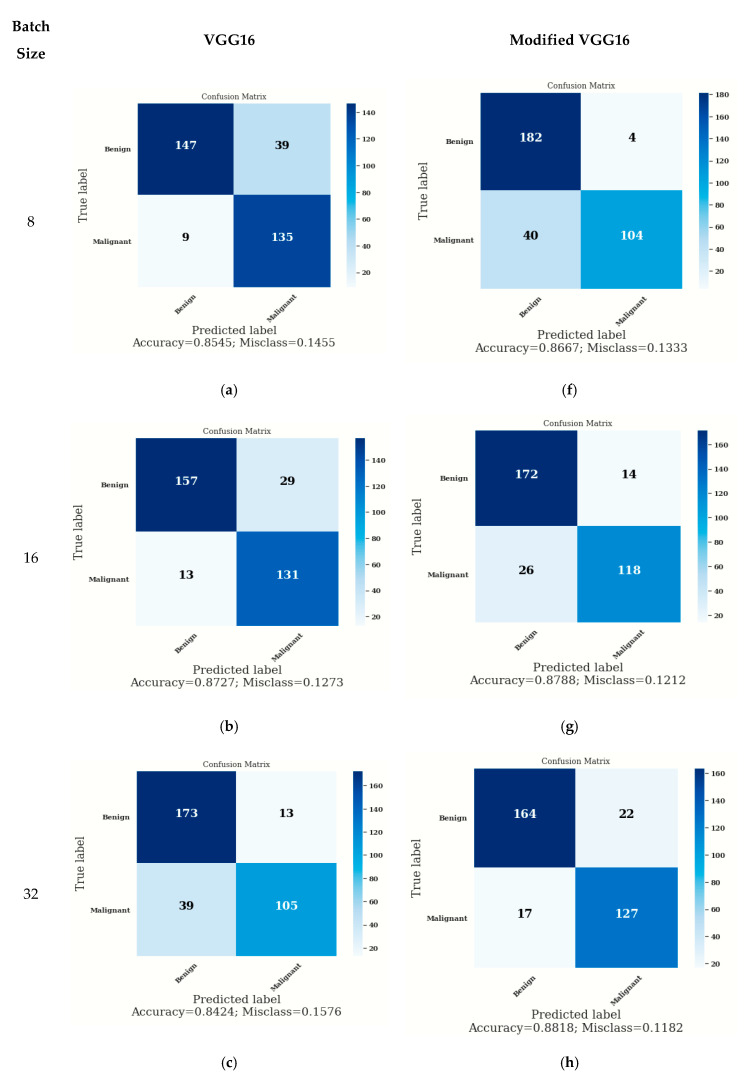

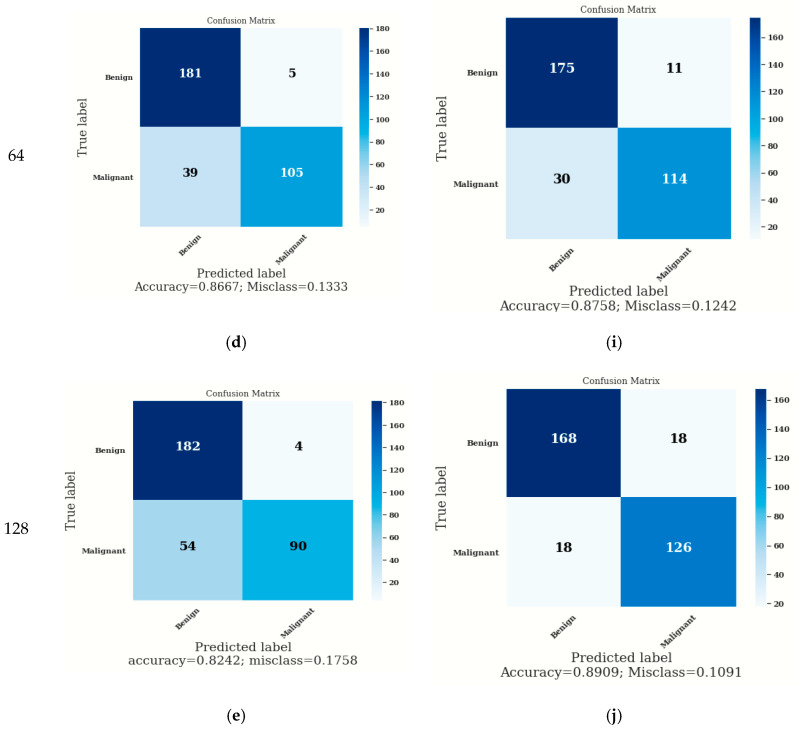
Confusion Matrix for VGG16 on Batch Size (**a**) 8 (**b**) 16 (**c**) 32 (**d**) 64 (**e**) 128, Confusion Matrix for Modified VGG16 on Batch Size (**f**) 8 (**g**) 16 (**h**) 32 (**i**) 64 (**j**) 128.

The confusion matrix provided predictions of true and false values as shown in Figure 8. True labels are indicated vertically, and predicted labels are indicated horizontally from which False Negatives (FN), False Positives (FP), True Positives (TP), and True Negatives (TN) can be calculated. The parameter accuracy is calculated using TP, TN, FP, and FN as given in Equation (3)

(3) $$\mathrm{Accuracy}=\frac{\mathrm{TP}+\mathrm{TN}}{\mathrm{TP}+\mathrm{FN}+\mathrm{FP}+\mathrm{TN}}\;$$

Figure 8a–e shows the confusion matrix for VGG16, and Figure 8f–j shows the confusion matrix for modified VGG16. In the case of modified VGG16, the values of accuracy are higher in comparison to the VGG16 model. On batch size 8, the accuracy on the modified VGG16 model is 86.67%, whereas, on the VGG16 model, it is 85.45%. On batch size 16, the accuracy is approximately similar to the VGG16 model and the modified VGG16 model as shown in Figure 8b,g. with batch size 32, the accuracy on the modified VGG16 model is better (i.e., 88.18%), whereas, on the VGG16 model, it is 84.24%. On batch size 64, the accuracy on the modified VGG16 model is 87.58%, whereas, on the VGG16 model, it is 86.67%. On batch size 128, the accuracy on the modified VGG16 model is 89.09%, whereas, on the VGG16 model, it is 82.42%. From Figure 8, it can be concluded that the modified VGG16 model is showing better accuracy as compared to VGG16 for each batch size (i.e., 8, 16, 32, 64, and 128).

### 3.4. Confusion Matrix Parameter Analysis

The confusion matrix parameter values are calculated by using Equations (4), (5), and (6), respectively.

(4) $$\mathrm{Precision}=\frac{\mathrm{TP}}{\mathrm{TP}+\mathrm{FP}}\;$$

(5) $$\mathrm{Sensitivity}=\frac{\mathrm{TP}}{\mathrm{TP}+\mathrm{FN}}\;$$

(6) $$F1\;\mathrm{Score}=\frac{2\times \mathrm{TP}}{2\times \left(\mathrm{TP}+\mathrm{FP}+\mathrm{FN}\right)}\;$$

**Figure 9:**
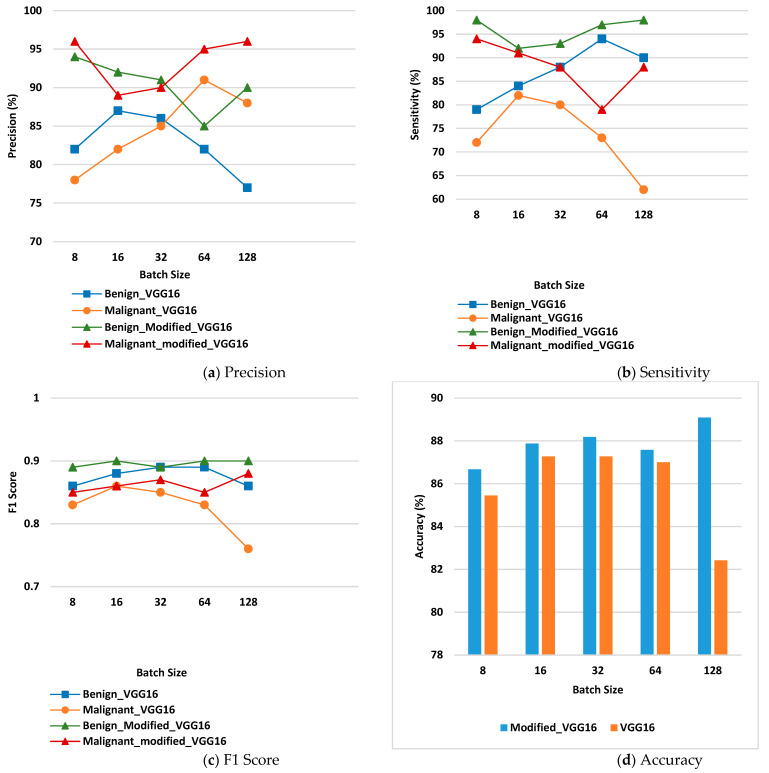
Confusion Matrix Parameters (**a**) Precision, (**b**) Sensitivity, (**c**) F1 Score, (**d**) Accuracy.

Figure 9 displays the values of confusion matrix parameter analysis for the benign and malignant class. Figure 9a displays precision for benign and malignant disease class on the VGG16 model and the modified VGG16 model. From the figure, it can be seen that the values of precision are higher in case of the modified VGG16 model at each batch size for benign as well as malignant class. For benign class, modified VGG16 is working best for batch size 8, whereas, for malignant class, it is working best for batch size 8 and 128.

Figure 9b shows the values of sensitivity for the benign and malignant disease classes on the VGG16 model and the modified VGG16 model. From the figure, it can be seen that the values of sensitivity are higher in the case of the modified VGG16 model at each batch size for benign as well as malignant class. For benign class, modified VGG16 is working best for batch size 8 and 128 whereas, for malignant class, it is working best for batch size 8 and 16.

Figure 9c shows the values of F1 score for VGG16 and the modified VGG16 model. The values of F1 Score are higher in the case of the modified VGG16 model at each batch size for benign as well as malignant class as compared to the VGG16 model. For benign class, modified VGG16 is working best for batch size 16, 64 and 128 whereas; for malignant class, it is working best for batch size 128.

Figure 9d shows the accuracy values of benign and malignant disease class on VGG16 and the modified VGG16 model. From the figure, it is detected that the overall ac-curacy is high in the case of the modified VGG16 model at each batch size as compared to the VGG16 model. Modified VGG16 is working best for batch size 128 in terms of overall accuracy, and the value is 89.09%.

### 3.5. Comparison with the State-of-the-Art

A comparison with other state-of-the-art methods has been performed using skin dermoscopy images in terms of accuracy and is presented in Table 7. The result analysis shows that the presented model has achieved good accuracy as compared to other state-of-the-art models. The accuracy is different in all studies, as different datasets (HAM10000, Kaggle and clinical images) are used. With HAM10000 dataset, Khan et al. [8] achieved an accuracy of 80.46% using VGG16 model architecture, and Agrahari et al. [10] and Chaturvedi et al. [11] had achieved accuracy rates of 80.81% and 83.10%, respectively on MobileNet model architecture.

## 4. Conclusions

The modified VGG16 model is trained using transfer learning. The training and testing of the model is performed on the Kaggle dataset. The presented model has been analyzed with various batch sizes of 8, 16, 32, 64, and 128 using the Adam optimizer and 10 Epochs. The proposed model is working best with overall accuracy of 89.09% on 128 batch size with Adam optimizer and 10 epochs. There is still a scope in improving the overall accuracy of the presented model. It can be enhanced by increasing both true positives as well as true negatives simultaneously. There is always a possibility to build a more suitable model for detection of skin cancer.

## Figures and Tables

**Table 1:** Dataset Description.

	Total	Training	Test	Validation
Benign	1800	1534	186	80
Malignant	1497	1284	144	69
Total	3297	2818	330	149

**Table 2:** Dataset Description of training images.

	Before Augmentation	After Augmentation
Benign	1534	3068
Malignant	1284	2568
Total	2818	5636

**Table 3:** Filtered Images after Convolution and Maxpool Layer.

	Conv-1	Conv-2	Conv-3	Max Pool
Block 1	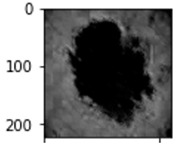	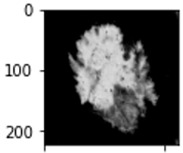	NA	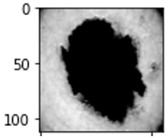
Block 2	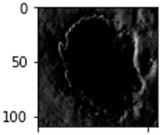	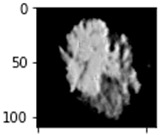	NA	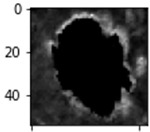
Block 3	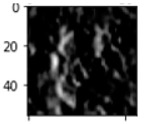	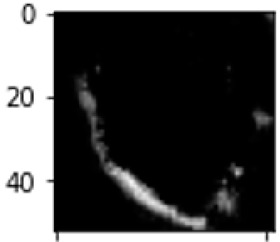	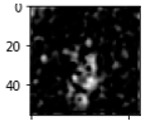	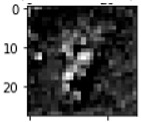
Block 4	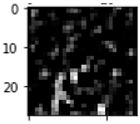	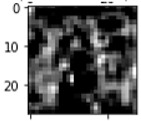	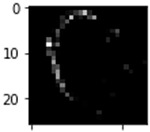	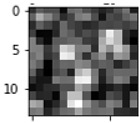
Block 5	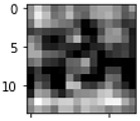	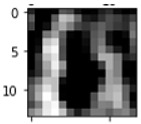	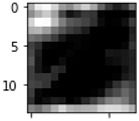	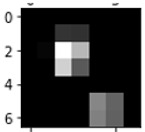

**Table 4:** Parameters of Proposed Model.

Layer	Shape of Output	Parameters
VGG16	7, 7, 512	1,471,468
Flatten	25,088	0
Dense_1	32	802,848
LeakyReLU_1	32	0
Dense_2	16	528
LeakyReLU_2	16	0
Dense_3	1	17
Total Parameters		15,518,081
Trainable Parameters		803,393
Non-trainable Parameters		14,714,688

**Table 5:** Hyper Tuning Parameters.

S. No.	Parameter	Value
1.	Batch size	8, 16, 32, 64, 128
2.	Optimizer	Adam
3.	Epochs	20

**Table 6:** Training Performance of Modified Vgg16 Model with Adam Optimizer.

Epoch Value	Batch Size	Train Loss	Train Accuracy	Validation Loss	Val Accuracy (%)
10	8	0.1912	0.9150	0.4346	83.22
	16	0.1705	0.9246	0.4664	83.89
	32	0.1736	0.9255	0.4310	81.88
	64	0.1957	0.9168	0.4034	83.22
	128	0.2293	0.8971	0.3514	84.56
20	8	0.1077	0.9510	1.0940	82.55
	16	0.0876	0.9645	0.7804	81.88
	32	0.0801	0.9698	0.7754	81.88
	64	0.0944	0.9634	0.6499	80.54
	128	0.1133	0.9546	0.6468	79.87

**Table 7:** Comparison of the Proposed Model with State-of-the-Art Techniques.

Ref	Technique Used	Dataset	Accuracy (%)
Khan et al. [8]	VGG16	HAM10000	80.46
Agrahari et al. [10]	MobileNet	HAM10000	80.81
Chaturvedi et al. [11]	MobileNet	HAM10000	83.10
Abdar et al. [13]	Bayesian Deep Learning Method	Kaggle	88.95
Fjisawa et al. [14]	Deep Convolutional Neural Network	Clinical Images	76.50
Garcia et al. [15]	Machine Learning	Interactive Atlas of Dermoscopy	88.00
Hasan et al. [38]	CNN	Kaggle	89.5
Singh et al. [39]	ResNet50	Kaggle	80.3
**Proposed**	**Modified VGG16 architecture**	**Kaggle**	**89.09**

## Data Availability

The data presented in this study are available on request from the corresponding author.
